# Clinical Features and Changes in Insulin Requirements in People with Type 2 Diabetes Requiring Insulin When Hospitalised with SARS-CoV-2 Infection

**DOI:** 10.1155/2022/8030765

**Published:** 2022-02-26

**Authors:** Antonella Corcillo, Aaisha Saqib, Niruthika Sithamparanathan, Amina Khanam, Jamal Williams, Abhiti Gulati, Dulmini Kariyawasam, Janaka Karalliedde

**Affiliations:** ^1^School of Cardiovascular Medicine and Sciences, King's College London, 3.11 Franklin-Wilkins Building, Waterloo Campus, Stamford Street, London SE1 9NH, UK; ^2^Guy's and St Thomas NHS Foundation Trust, Guy's Hospital, Great Maze Pond, London SE1 9RT, UK

## Abstract

**Background:**

Uncontrolled hyperglycaemia before and during hospitalisation is a risk factor for adverse outcomes in people with diabetes and SARS-CoV-2 infection. Insulin often at high doses is frequently required to manage hyperglycaemia associated with SARS-CoV-2 infection during hospitalisation. However, there is limited information on the clinical features and sequelae of people with type 2 diabetes (T2DM) not previously on insulin that require insulin as a new treatment when hospitalised with SARS-CoV-2 infection.

**Aims:**

To describe the clinical features and insulin treatment sequelae of 113 people with T2DM that required insulin as a new treatment when hospitalised with SARS-CoV-2 infection.

**Methods:**

A single-centre study of 113 people with T2DM who were not on insulin before their admission for SARS-CoV-2 infection. The primary aim of our study was to identify clinical and biochemical features that were associated with the need for insulin as a new treatment in people with known T2DM not on insulin treatment at the time of hospitalisation for SARS-CoV-2 infection. We also describe changes in insulin requirements at time of discharge from hospital and 6 weeks later during the first wave of SARS-CoV-2 infection (April–March 2020) in the UK. Clinical, biochemical, and anthropometric data were collected from electronic health records.

**Results:**

We observed that of 113 people with T2DM, 35% (*n* = 39) needed insulin as a new treatment during their hospitalisation for SARS-CoV-2 infection. People requiring insulin were younger, had a higher preadmission HbA1c, were more frequently on oral medication for diabetes before the admission, and were more likely to be obese (body mass index ≥30 kg/m^2^), with *p* ≤ 0.001 for all. In multivariable logistic regression analyses, we observed that younger age and higher HbA1c before admission were independently associated with needing insulin, with one-year increase in age associated with decreased odds of needing insulin initiation (OR 0.91, 95% CI 0.83–0.99), and increasing preadmission HbA1c by 1 mmol/mol associated with an increased odds of insulin initiation (OR 1.05, 95% CI 1.002–1.11) (*p* < 0.05 for both). Of the 39 people with T2DM who required insulin as a new treatment, 28% remained on insulin at the time of discharge with their insulin dose falling from 1.26 U/kg within the first 7 days of admission to 0.39 U/kg at discharge. At 6 weeks after discharge, 24% of people remained on insulin.

**Conclusion:**

More than one-third of people with T2DM not previously treated with insulin required new insulin treatment when hospitalised with SARS-CoV-2 infection, and of this group, 24% remained on insulin at 6 weeks after discharge. This study highlights the important variations of insulin requirements in people with T2DM new to insulin and the importance of a dedicated team for patient education and close follow-up.

## 1. Introduction

Diabetes has been associated with a higher risk of critical care unit admission and mortality in patients admitted with SARS-CoV-2 infection [1, 2]. Direct effects of SARS-CoV-2 on the pancreas have been previously described [3–7]. Hyperglycemia and ketonemia induced by direct impairment of ß-cell function and transdifferentiation of ß-cells showing reduced insulin secretion have been reported [3–7]. This bidirectional relationship between SARS-CoV-2 and diabetes, where, on the one hand, diabetes represents a risk factor for more severe SARS-CoV-2 infection outcomes and on the other hand, SARS-CoV-2 has direct effects on the pancreas, may lead to reduced insulin release [4]. Suboptimal glycaemic control before and during hospitalisation is a risk factor for adverse outcomes in people with diabetes and SARS-CoV-2 infection [8–12]. Many meta-analyses have reported a 2- to 3-fold increased risk of severe SARS-CoV-2 infection and mortality in people with diabetes [13–16]. Compared to people without diabetes, people with diabetes have been reported to have higher mortality [17]. Also, an elevated glycated haemoglobin (HbA1c >9%) has been associated with a nearly 60% increased risk of hospitalisation for SARS-CoV-2 pneumonia [18, 19]. Insulin often at high doses is frequently required to manage hyperglycaemia associated with severe SARS-CoV-2 infection in people with diabetes. High dose requirements of insulin during the admission for severe SARS-CoV-2 infection have been previously described [20–22].

In a recent Italian cohort of 551 patients with SARS-CoV-2 infection and without any preexisting known history of diabetes, among 46% of the patients with hyperglycaemia during the admission, glycaemic abnormalities were found in the participants at least 2 months after recovery [23]. However, to our knowledge, there is little information on the clinical features and sequelae of people with diabetes that require insulin when admitted with SARS-CoV-2 infection.

The primary aim of our study was to identify clinical and biochemical features that were associated with the need for insulin as a new treatment in people with known type 2 diabetes (T2DM) not on insulin treatment at the time of hospitalisation for SARS-CoV-2 infection and to describe changes in insulin requirements at the time of discharge from the hospital and 6 weeks later during the first wave of SARS-CoV-2 infection (April–March 2020) in the UK.

A secondary aim was to compare the clinical features and insulin treatment sequelae/requirements between the first and second wave of SARS-CoV-2 infection (December 2020–January 2021) in people with T2DM who required insulin as a new treatment.

## 2. Materials and Methods

For our primary aim, we retrospectively collected data from 113 consecutive people with known T2DM who were not on insulin before their admission for SARS-CoV-2 infection at one hospital, during the 1st (April-March 2020) wave of SARS-CoV-2 infection in the UK. All patients had a positive polymerase chain reaction SARS-CoV-2 swab test. Insulin requirements during hospitalisation and at 6 weeks after discharge from the hospital were evaluated. Clinical, biochemical, and anthropometric data were collected from electronic health records. Inclusion criteria were people with known T2DM not on insulin treatment. People with type 1 diabetes, with gestational diabetes, with T2DM on insulin prior to admission, and with corticosteroids- or stress-induced hyperglycaemia were excluded from the study. Criteria for SARS-CoV-2 infection that required hospitalisation included severe shortness of breath at rest or difficulty breathing, reduced oxygen saturation levels measured by pulse oximetry, and clinical features of respiratory distress. All people hospitalised required oxygen support. The need for insulin and insulin requirements during hospitalisation and at 6 weeks after discharge from the hospital were evaluated. Multivariable logistic regression analyses were performed in this group to determine variables associated with the need for insulin treatment.

For our secondary aim, further analyses were done to compare the insulin treatment sequelae between the first wave and the second wave of SARS-CoV-2 infection (when use of dexamethasone was the standard of care for SARS-CoV-2 in London). In this analysis, we evaluated data from 43 people with known T2DM, not on insulin, admitted to the hospital for SARS-CoV-2 during the 2nd (December 2020–January 2021) wave of SARS-CoV-2 infection but who required insulin as a new treatment due to their SARS-CoV-2 infection. We compared their insulin requirements and insulin treatment sequelae to those admitted during the 1st wave who required insulin as a new treatment (*n* = 39).

This study was performed as a service evaluation of our diabetes clinical service during the COVID-19 pandemic. No ethical approval was required for this study, as this was a service evaluation and clinical audit. All participant data were anonymised for analysis.

Descriptive statistics were used for the analysis of demographic and clinical features. Data are presented as *n* (%) or median (range). HbA1c was considered as a continuous variable, and body mass index (BMI) was defined as a binary outcome with a cutoff of 30 kg/m^2^. Data were compared using an unpaired *t*-test (for continuous normally distributed variables), Mann–Whitney test (for continuous variables not normally distributed), and *χ*^2^ test (for categorical variables). Multivariable logistic regression analyses were performed (only on the data set for the primary aim) to determine variables associated with the need for insulin treatment. Odds ratio and 95% confidence intervals are reported. A two-tailed *p* value < 0.05 was considered significant. Statistical analyses were performed with Stata/SE 15.0 (StataCorp LLC, TX, USA).

## 3. Results

Of the cohort of 113 people, 58% were men, with a median age of 68 years (range 42–97 years), a median duration of diabetes of 10 years (range 0–57 years), and a median HbA1c of 54 mol/mmol (range 33–137 mmol/mol) (7.1% (range 5.2–14.7%)). Prior to admission, 69% were on oral agents and 31% on diet only. Insulin was started in 35% of this cohort as a new treatment during admission, and in this group, 82% were already on oral medication for diabetes before their admission, compared to 62% who did not need insulin (Table 1). Median insulin requirements were 1.26 units/kilograms (U/kg) (range 0.15–4.5 U/kg) within the first 7 days of admission, 0.39 U/kg (range 0.06–0.84 U/kg) at discharge, and 0.23 U/kg (range 0.09–0.74 U/kg) 6 weeks after discharge, respectively. Of those on insulin, 28% and 24% were still on insulin at discharge and at 6 weeks after discharge, respectively. Table 1 shows baseline features between patients requiring initiation of insulin and those who did not need insulin during hospitalisation for SARS-CoV-2 infection. People requiring insulin were younger, had a higher HbA1c, were more frequently on oral medication for diabetes before the admission, and were more frequently obese (BMI ≥30 kg/m^2^), with *p* ≤ 0.001 for all. There were no differences in gender, ethnicity, duration of diabetes, comorbidities, or mortality. We performed multivariable logistic regression analyses where preadmission HbA1c, age, oral medication, and BMI ≥30 kg/m^2^ were entered into the model. We observed that one-year increase in age was associated with decreased odds of 9% in insulin requirements (OR 0.91, 95% CI 0.83–0.99), while increasing HbA1c by 1 mmol/mol was associated with an increased odds of insulin requirement of 5% (OR 1.05, 95% CI 1.002–1.11).

In the second wave of SARS-CoV-2 infection in the UK, when corticosteroids were the established treatment for SARS-CoV-2 infections, in secondary analyses, we compared the features of people with known T2DM who required insulin as a new treatment in the first wave of SARS-CoV-2 infection (*n* = 39) in April–March 2020 in the UK with those with known T2DM who also required insulin as a new treatment when hospitalised in the second wave of SARS-CoV-2 infection (*n* = 43) (December 2020–January 2021).

When comparing the group from the 1st wave (*n* = 39) to the group from the 2nd wave (*n* = 43) who needed insulin as a new treatment during their admission, people from the 2nd wave were less likely to be of Afro-Caribbean ethnicity (26% vs 52%), had a lower prevalence of chronic kidney disease (5% vs 21%), and had less retinopathy (5% vs 43%) (all *p* < 0.03). People who needed insulin in the 2nd wave also had a shorter duration of hospitalisation (8 vs 22 days, *p* < 0.001) as compared to those in the 1st wave.  Table 2 shows the insulin requirements and changes in insulin doses at discharge and after 6 weeks during the 1st and the 2nd waves.

The percentage of people still on insulin at discharge was higher during the second wave (56% vs 28%, *p* = 0.03). However, at 6 weeks after discharge, it was similar in both groups (24% vs 22%, *p* = 0.87). Insulin requirements were also similar at discharge (0.39 U/kg vs 0.34 U/kg, p 0.98) and at 6 weeks after discharge (0.23 U/kg vs 0.31 U/kg, p 0.37) between people in the first and second wave who needed insulin.

## 4. Discussion

Our results from 113 people with known T2DM not on insulin treatment at the time of hospitalisation for SARS-CoV-2 infection between April and March 2020 demonstrate that 35% of people with known diabetes required insulin as a new treatment during their hospitalisation. Moreover, their insulin requirements change dynamically during hospitalisation and at discharge. In our secondary analyses, we observed that changes in insulin requirements were more pronounced in people admitted with SARS-CoV-2 infection in the second wave, most likely due to more prevalent use of corticosteroid treatment for SARS-CoV-2 infection. In a recent Italian study, persistent insulin resistance and *β*-cell dysfunction were demonstrated during admission for SARS-CoV-2 infection [23]. The use of dexamethasone has been shown to lower 28-day mortality in patients admitted with SARS-CoV-2 infection who were receiving either invasive mechanical ventilation or oxygen alone at randomization compared to those receiving no respiratory support [24, 25]. Therefore, dexamethasone was largely prescribed in patients admitted to the hospital during the 2nd wave. Glucocorticoids-induced hyperglycaemia is a well-known complication. The main mechanisms leading to hyperglycaemia are increased insulin resistance and glucose production via hepatic gluconeogenesis, and inhibition of insulin production and secretion by pancreatic ß-cells [26, 27]. Direct effect of SARS-CoV-2 on the pancreas, “stress-induced hyperglycaemia” and hyperglycaemia secondary to treatments such as glucocorticoids, has been previously charged as potential underlying reasons associated with hyperglycaemia in patients infected with SARS-CoV-2 [28].

Poorly controlled glycaemic control has been linked to more severe SARS-CoV-2 infection outcomes. In a cohort of 810 patients with diabetes, Zhu et al. described a significant increase in septic shock, acute kidney injury and acute heart injury in patients with poorly controlled diabetes defined as blood glucose >180 mg/dl, compared to well-controlled patients with diabetes [8].

In another retrospective observation study, Bode et al. reported a significantly higher risk of death in patients with SARS-CoV-2 infection who presented uncontrolled hyperglycaemia (defined as 2 blood glucose value, >180 mg/dl within any 24-hour period) but were not diagnosed as people with diabetes (HbA1c < 6.5%), compared to patients with known diabetes [10]. This suggests a major role of stress-induced hyperglycaemia in poorer outcomes [29].

The impact of SARS-CoV-2 on pathways that drive insulin resistance has been described, and this may explain the raised insulin requirements in the acute phase of the infection by SARS-CoV-2 and why this may fall as the infection resolves with subsequent clinical improvement [30, 31].

In our study of people from the first wave of SARS-CoV-2 (December 2020–January 2021), more than half of the people who needed insulin during their hospitalisation remained on insulin at the time of discharge, and at 6 weeks after discharge more than 20% still required insulin treatment. Our hospital had developed a care pathway where people leaving the hospital after SARS-CoV-2 infection with insulin as a new treatment received weekly phone calls and advice by a multidisciplinary team, with guidance on close monitoring of home capillary blood glucose levels and education on recognition and self-management of hypoglycaemia. A recent study showed that in a cohort of 660 adults hospitalised with suspected SARS-CoV-2 pneumonia, the prevalence of dysglycaemia resolved to preadmission frequency after the infection resolved [23]. However, in this cohort, only 19.6% of participants had preexisting or newly diagnosed diabetes [23].

There is a consensus that supports the use of insulin for people with diabetes who are hospitalised with SARS-CoV-2 infection [32–36]. Optimal glucose control with insulin can reduce inflammatory markers and prognostic indices such as IL-6 and D-dimer levels and clinical outcomes. There is limited data on oral antidiabetic agents in SARS-CoV-2 infections. Metformin has shown anti-inflammatory activity in preclinical studies, and a study in patients with T2DM and SARS-CoV-2 that compared those receiving metformin to those who did not demonstrated reduced in-hospital mortality [37, 38]. However, it is likely this is due to selection bias, as severely ill patients could not receive metformin [37,38].

The strengths of our study were the single-centre cohort with standardised treatment pathways during hospitalisation and at discharge. We also collected follow-up data after discharge and reported data from two cohorts (before and after use of corticosteroids for SARS-CoV-2 infection in people with diabetes who required insulin as a new treatment for managing their diabetes during hospitalisation). The limitations of our study were the relatively small size of the cohort from a single centre, the retrospective study design, the inclusion of only people with T2DM hospitalised with SARS-CoV-2 infection with exclusion of people who did not require admission to hospital for their infection, and the relatively short follow-up period after discharge.

## 5. Conclusion

In conclusion, our results highlight that treatment with insulin is frequently needed in people with T2DM with SARS-CoV-2 infection and emphasise the importance of a dedicated multidisciplinary team to provide education and self-management of insulin and guide and support people with diabetes on self-adjusting insulin doses after discharge home.

## Figures and Tables

**Table 1:** Baseline clinical features of people with known type 2 diabetes who required initiation of insulin as a new treatment to those who did not when hospitalised with SARS-CoV-2 infection.

	Insulin treatment needed (*n* = 39)	Insulin treatment not needed (*n* = 74)	*p* value
Age, years	60 (43–86)	76 (42–97)	<0.001
Gender (male)	25 (64%)	40 (54%)	0.30
*Ethnicity*			
Caucasian	23 (42%)	9 (33%)	0.49
Afro-Caribbean	21 (38%)	14 (52%)	0.46
Other	11 (20%)	4 (15%)	0.24
Duration of diabetes, years	11 (2–29)	8 (0–57)	0.47
HbA1c at admission, mmol/mol	63 (34–137)	52 (33–81)	0.001
HbA1c at admission, %	7.9 (5.3–14.7)	6.9 (5.1–9.6)	
Oral medication for diabetes before admission	32 (82%)	46 (62%)	0.030
*Previous history*			
Hypertension	31 (79%)	54 (73%)	0.45
Chronic kidney disease ≥ stage 3	8 (21%)	24 (32%)	0.18
Cardiovascular disease	5 (13%)	20 (27%)	0.08
Cerebrovascular disease	6 (15%)	14 (19%)	0.64
Peripheral polyneuropathy	4 (10%)	8 (11%)	0.93
Retinopathy	16 (43%)	21 (33%)	0.29
Body mass index ≥30 kg/m^2^	22 (73%)	14 (31%)	<0.001
Mortality	11 (28%)	21 (28%)	0.98
Admission to intensive care unit	28 (72%)	8 (11%)	<0.001
Intubation	28 (72%)	5 (7%)	<0.001
Duration of hospitalisation, days	22 (1–101)	7 (1–54)	<0.001

Data are shown as *n* (%) or median (range). In the full cohort of 113 people, data are available for ethnicity (n = 82), duration of diabetes (n = 97), HbA1c at admission (n = 54), and body mass index (n = 75)..

**Table 2:** Comparison between the first (April–March 2020) and second wave of SARS-CoV-2 infection (December 2020–January 2021) of insulin requirements and changes of insulin doses (at discharge and after 6 weeks) in people with known T2DM who required insulin as a new treatment during hospitalisation for SARS-CoV-2 infection.

	1^st^ wave (*n* = 39)	2^nd^ wave (*n* = 43)	*p* value
Total daily dose at discharge, units/kg	0.39 (0.06–0.84)	0.34 (0.12–0.85)	0.98
% of people still on insulin at discharge	28%	56%	0.03
Total daily dose within 6 weeks of discharge, units/kg	0.23 (0.09–0.74)	0.31 (0.17–0.43)	0.37
% of people still on insulin within 6 weeks of discharge	24%	22%	0.87

Data are shown as % or median (range).

## Data Availability

Data are available on request to the corresponding author.
